# Z-DNA Hunter tool for straightforward detection of Z-DNA forming regions and a case study in *Drosophila*

**DOI:** 10.1093/nargab/lqaf166

**Published:** 2025-11-21

**Authors:** Michal Petrovič, Martin Bartas, Alistair N Garratt, Petr Pečinka, Michaela Dobrovolná, Klára Koňaříková, Oldřich Trenz, Václav Brázda, Jiří Šťastný

**Affiliations:** Department of Informatics, Mendel University in Brno, Zemědělská 1, 613 00 Brno, Czech Republic; Department of Biology and Ecology, University of Ostrava, 710 00 Ostrava, Czech Republic; The Brainstem Group, Institute for Cell Biology and Neurobiology, Center for Anatomy, Charité University Hospital, 10117 Berlin, Germany; Department of Biology and Ecology, University of Ostrava, 710 00 Ostrava, Czech Republic; Institute of Biophysics of the Czech Academy of Sciences, Královopolská 135, 612 00 Brno, Czech Republic; Faculty of Chemistry, Brno University of Technology, Purkyňova 118, 612 00 Brno, Czech Republic; Institute of Biophysics of the Czech Academy of Sciences, Královopolská 135, 612 00 Brno, Czech Republic; Faculty of Chemistry, Brno University of Technology, Purkyňova 118, 612 00 Brno, Czech Republic; Department of Informatics, Mendel University in Brno, Zemědělská 1, 613 00 Brno, Czech Republic; Institute of Biophysics of the Czech Academy of Sciences, Královopolská 135, 612 00 Brno, Czech Republic; Faculty of Chemistry, Brno University of Technology, Purkyňova 118, 612 00 Brno, Czech Republic; Department of Informatics, Mendel University in Brno, Zemědělská 1, 613 00 Brno, Czech Republic

## Abstract

Z-DNA is a left-handed DNA conformation linked to gene regulation, chromatin dynamics, and immunity. Despite its importance, genome-wide prediction of Z-DNA forming sequences (ZFS) remains limited by the absence of fast and accessible tools. Here, we present Z-DNA Hunter, a user-friendly web server for genome-scale ZFS prediction utility. The algorithm employs a pattern-based approach optimized for canonical motifs such as (GC)n and (CA)n repeats, with adjustable parameters for detection stringency. Compared with existing methods, Z-DNA Hunter achieves similar or higher accuracy while reducing runtime from hours to seconds, making large-scale analyses feasible. To demonstrate its application, we analyzed the *Drosophila melanogaster* genome and uncovered a pronounced enrichment of long ZFS on the X chromosome, contrasting with their near absence on the satellite repeat- and transposable element-rich Y chromosome. These findings illustrate both the scalability of Z-DNA Hunter and its potential to reveal biologically meaningful patterns of non-B-DNA. The tool provides direct visualization and export options (e.g. BedGraph for UCSC Genome Browser) and is freely available at https://bioinformatics.ibp.cz/#/analyse/zdna.

## Introduction

Z-DNA is a left-handed form of DNA discovered thanks to the pioneering work of Fritz Pohl and Thomas Jovin, published in 1972 [1]. Later, in 1979, Alexander Rich’s team successfully crystallized Z-DNA [2]. For many years, Z-DNA was viewed as a mere structural anomaly, lacking any significant molecular or biological functions. It is now understood that Z-DNA primarily forms in specific regions known as Z-DNA forming sites (ZFS), which are characterized by alternating pyrimidine and purine bases. Common examples of these sites include sequences like (GC)n and (GT)n/(CA)n. The key requirement for Z-DNA formation is the regular alternation of pyrimidines and purines, which adopt, respectively, an alternating pattern of anti- and syn-configurations of sugar-base N-glycosidic bonds, creating a zig-zag-shaped sugar phosphate backbone that prefers a left-handed conformation under certain conditions. However, it has been found that some G+C content is necessary for Z-DNA to form; in other words, pure (AT)n repeats typically do not lead to Z-DNA formation [3]. The formation of Z-DNA is influenced by several environmental and biochemical factors, including supercoiling stress [4], high salt concentrations [1, 5], interactions with certain proteins [6], and cytosine methylation [7]. Thus, Z-DNA is a highly dynamic structure in living cells, whereby the switch from B- to Z-DNA conformation at Z-DNA forming sites is regulated in a cell- and tissue-specific manner that can be influenced by extrinsic and intrinsic signaling cues [8, 9, 10]. Under physiological conditions, negative supercoiling generated during active transcription can transiently stabilize Z-DNA structures, particularly in promoter regions or near transcriptional start sites [11]. This dynamic formation suggests a potential regulatory role in gene expression. Furthermore, proteins such as ADAR1, which contains a Zα domain capable of specifically recognizing and binding Z-DNA [12], lends support to the hypothesis that Z-DNA is not merely a structural curiosity but rather a functional element involved in RNA editing, immune signaling [6, 13], and possibly genome stability [14].

Recent studies using genome-wide mapping techniques have identified Z-DNA-forming regions across various species, including humans [11]. The first identification of the Z-DNA conformation in material of biological origin was through use of anti-Z-DNA antibodies in insects, including the interband regions of *Drosophila* polytene chromosomes [15] and *Chironomus* [16]. These findings suggest that Z-DNA tends to form in actively transcribed regions of the genome [17], highlighting its evolutionary conservation and functional relevance. ZFS are often enriched near genes involved in immune response [18], apoptosis [19], and cell cycle regulation [20, 21, 22], raising intriguing questions about their roles in stress responses [23, 24], disease pathogenesis [25], including neurological [26] and genetic disorders [27], and cancer [28]. Notably, some viruses also appear to exploit Z-DNA or its recognition machinery [29], potentially modulating host gene expression to their advantage.

Collectively, growing evidence has shifted the view about Z-DNA from an exotic structural form to a dynamic regulatory element with important biological functions. Understanding how DNA sequence and chromatin context influence Z-DNA formation is essential for revealing its roles in both normal physiology and disease. In this context, bioinformatics tools are crucial for identifying and characterizing ZFS across the genome. Therefore, we developed DNA Analyser, which is a web-based platform that allows users to detect and classify DNA segments capable of forming local nucleic acid structures. The Z-DNA Hunter module within the platform uses two classification models that assess sequence features, particularly pyrimidine–purine dinucleotides such as GC, TG/CA, or mixed purine–pyrimidine tracts, to predict the likelihood of Z-DNA formation as these have previously been shown to be capable of forming Z-DNA structures [30, 31]. Users can input nucleotide sequences in FASTA format, which are then processed using algorithms that evaluate Z-forming potential in a few seconds, even for large eukaryotic chromosomes. Results are presented through clear graphs and tables that indicate the position, length, and probability of local structure formation. The platform also supports manual parameter adjustments and provides user-friendly visual outputs to help interpret results and locate regions with possible regulatory or structural significance.

## Implementation

**Figure 1.**
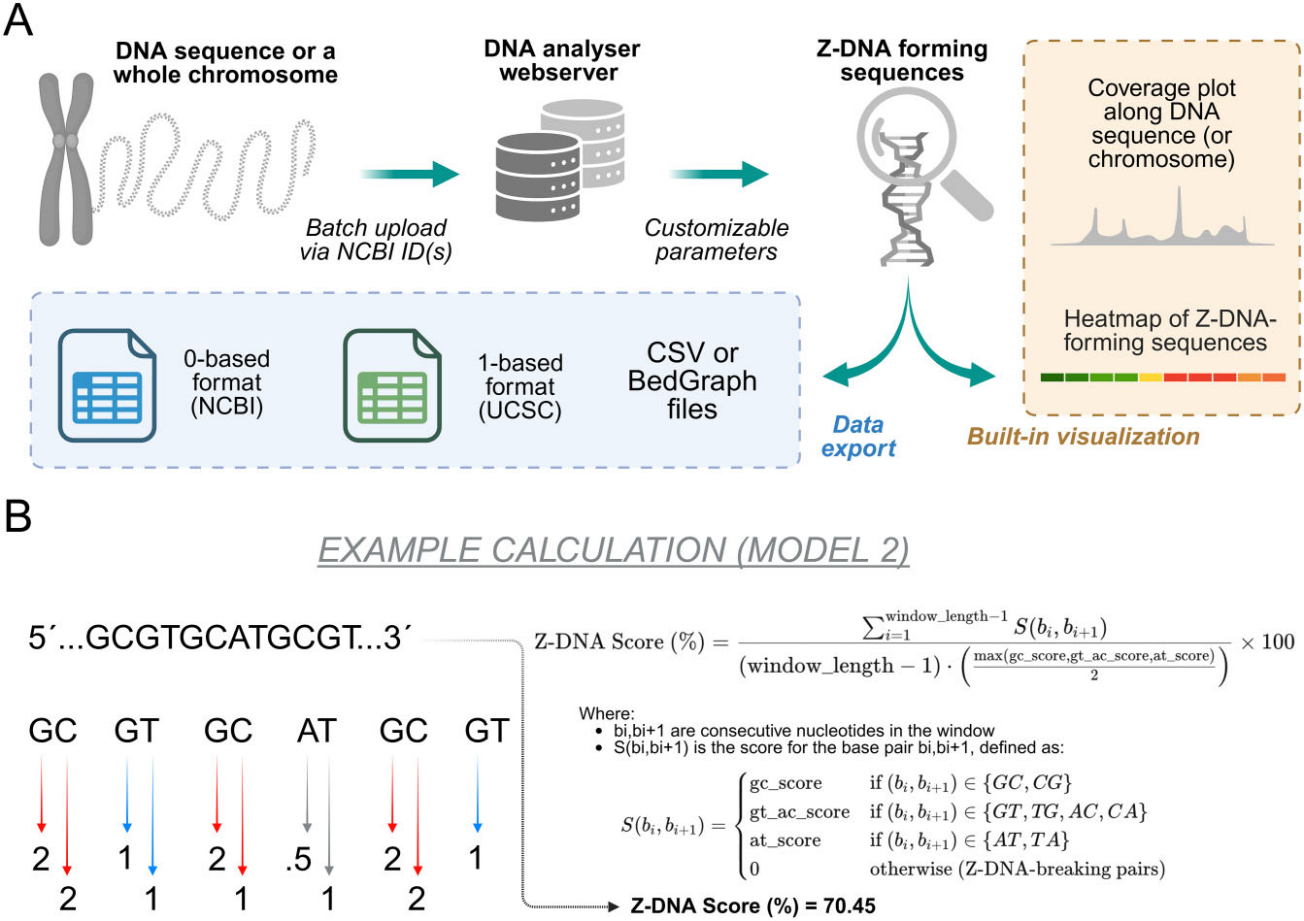
Z-DNA Hunter program. (**A**) Schematic depiction of Z-DNA Hunter analysis workflow and possible data and visualization outputs. (**B**) Calculation of Z-DNA score for an example Z-forming sequence with *Model 2* scoring system.

Web application output: Z-DNA Hunter uses an interactive web interface with Asynchronous JavaScript and XML (AJAX) to update dynamically its display of analysis results. Along with comprehensive statistics and sequence characteristics, it displays a heatmap of the ZFS distribution and allows multiple simultaneous analyses, each in its own tab. The results can be exported in BedGraph and CSV formats for further study or record keeping. A schematic depiction of the Z-DNA Hunter workflow is depicted in Fig. 1A. The information provided for each identified ZFS includes:

Position and length: Precise genomic location information using genomic coordinates.
Sequence: The expandable nucleotide sequence of the ZFS for additional analysis.
Z-DNA GC richness: The GC dinucleotide content within the ZFS, which correlates with its stability and propensity for Z-DNA formation.
Z-DNA GT richness: The GT dinucleotide content within the ZFS, which can also influence Z-DNA formation.
Z-DNA score: The overall score is calculated based on the specific dinucleotides and their propensities for Z-DNA formation.
Z-DNA score (%): The raw Z-DNA score divided by the maximum possible score for that window, then multiplied by 100, indicating the relative propensity of the sequence to adopt a Z-DNA conformation.

Use of the API and export options: Z-DNA Hunter allows users to export results in formats compatible with genome browsers for visual analysis and the overlay of regions on genomic maps. The results can be downloaded in CSV format, which is compatible with many spreadsheet programs, and in BedGraph format, which works well with genome annotation tools, including the UCSC Genome Browser. The capabilities of Z-DNA Hunter are further expanded by the DNA Analyser API, which enables integration with unique scripts or web services for more extensive bioinformatics analyses and automated workflows.

Z-DNA Hunter development and integration: Z-DNA Hunter is part of a comprehensive suite of DNA sequence analysis tools developed by the research team. It is incorporated into the DNA Analyser web server, which integrates several complementary tools such as G4Hunter [32], Palindrome Analyser [33], and CpX Hunter [34]. The Z-DNA Hunter algorithm is based on the non-B-DNA Motif Search Tool [35]. The tool features a high-performance back-end and a user-friendly web interface to enable easy analysis and interactive visualization of results. All imported sequences and analyses are stored in a database for data persistence and future retrieval. An API is available in the web application to integrate Z-DNA Hunter with a wide set of sequence analysis tools. This facilitates batch processing and enables users to incorporate seamlessly Z-DNA analysis into their broader bioinformatics workflows.

Procedure for input and analysis: Z-DNA Hunter provides users with multiple options for inputting DNA sequences for analysis. They can directly upload files in FASTA or plain text format, use NCBI IDs to upload individual sequences, or upload DNA sequences in bulk directly from the NCBI Genome database. The web application also allows for direct clipboard input, enabling rapid testing of sequences. All uploaded sequences can be tagged for easy organization. The tool can accept files up to 2 GiB (gigibytes), corresponding to ∼2.1 billion bp, allowing for the analysis of whole chromosomes or substantial genomic regions. To fine-tune the identification of Z-DNA regions, users can customize various search parameters:

Minimal sequence size: Set by default to 12 bp to identify ZFS with at least one complete Z-DNA turn.
Score GC: The score for the GC dinucleotide, which particularly favors the formation of the Z-DNA structure. The default value is set to 25 for *Model 1* and 2 for *Model 2*.
Score GT/CA: The default value is set to 3 for *Model 1* and 1 for *Model 2*.
Score AT: The default value is set to 0 for *Model 1* and 0.5 for *Model 2*.
Minimal Score Percentage: The minimum score threshold for the searched ZFS.
These default parameter settings are based on previous experimental studies and can be adjusted by users to suit their specific analysis needs. Basically, *Model 2* is considered to be less strict, giving more weight to ZFS with mixed dinucleotide character (with respect to *Model 1*) and also positively scoring AT dinucleotides (which can be useful for rescuing some types of mixed sequences, e.g. GCGTGCATGTGTGC).

Methodology of detection: Z-DNA Hunter calculates scores based on the propensity of sequence regions to form the Z-DNA conformation. The scoring system operates on a linear principle, where each nucleotide receives a score based on defined parameters. This scoring process continues for each nucleotide until a dinucleotide is encountered that is rated 0 according to the parameters or one that cannot form Z-DNA regions (i.e. AA, CC, GG, TT, AG, CT, GA, and TC). At such a break in the sequence, the tool checks if the minimum window length and score thresholds are met. If both conditions are satisfied, the sequential window is marked as a potential ZFS. An example Z-DNA score calculation together with a general mathematical formula, is depicted in Fig. 1B. We also prepared a detailed Z-DNA Hunter Help Page, where additional information and example score calculations can be found: https://bioinformatics.ibp.cz/#/help/zdna.

Z-DNA Hunter provides a user-friendly interface for analyzing dinucleotide repeats and has significant potential to contribute valuable insights into the structural dynamics and biological functions of these unique DNA conformations. The research team expects this tool to help a wide range of researchers generate new hypotheses and facilitate exciting discoveries within the field of Z-DNA research.

## Case study—ZFS prediction in *Drosophila melanogaster* genome

In this study, we conducted an analysis of the occurrence and distribution of ZFS within the genome of the model organism *Drosophila melanogaster*. This species has a genome comprised of four pairs of chromosomes: three pairs of autosomes and one pair of sex chromosomes. The dm6 assembly represents these as five autosomal contigs (2L, 2R, 3L, 3R, and 4), along with two sex chromosomes (X and Y) and a mitochondrial genome. Because of its relatively simple genomic structure, *D. melanogaster* is an ideal candidate for clear and concise visualization. Using Z-DNA Hunter, we identified a total of 19 251 ZFS using Model 1 (with an average frequency of 0.140 ZFS per 1000 bp) and 35 907 ZFS with Model 2 (with an average frequency of 0.261 ZFS per 1000 bp). The original .bed files are included in Supplementary Material S1A and B. Basic statistics of ZFS occurrence (only ZFS with length at least 12 bp) in the *Drosophila* genome are depicted in Table 1 for particular chromosomes.

**Table 1.** Basic characteristics of ZFS in *Drosophila melanogaster*

	Characteristics	X	2L	2R	3L	3R	4	Y	MT
	Chr length (bp)	23 542 271	23 513 712	25 286 936	28 110 227	32 079 331	1 348 131	3 667 352	19 524
	G+C content (%)	42.5	42	42.5	41.5	42.5	35	39.5	18
Model 1	Count ZFS	5383	2869	3180	3213	4370	58	178	0
	Sum of ZFS (bp)	92 752	45 613	50 756	50 271	69 859	779	2374	0
	Median length ZFS (bp)	15	14	14	14	14	13	13	0
	Max ZFS length (bp)	58	71	83	119	103	21	21	0
	Freq ZFS per 1000 bp	0.229	0.122	0.126	0.114	0.136	0.043	0.049	0
	Coverage ZFS (%)	0.394	0.194	0.201	0.179	0.218	0.058	0.065	0
Model 2	Count ZFS	8225	5652	6289	6459	8650	186	446	0
	Sum of ZFS (bp)	135 790	85 612	96 271	97 368	131 454	2839	7158	0
	Median length ZFS (bp)	14	13	14	13	13	14	15	0
	Max ZFS length (bp)	66	71	85	119	103	26	21	0
	Freq ZFS per 1000 bp	0.349	0.240	0.249	0.230	0.270	0.138	0.122	0
	Coverage ZFS (%)	0.577	0.364	0.381	0.346	0.410	0.211	0.195	0

In columns, there are particular *Drosophila* chromosomes. Rows contain general characteristics (chromosome length and G+C content) and then specific characteristics regarding ZFS predicted either with “Model 1” or “Model 2” scoring systems.

**Figure 2.**
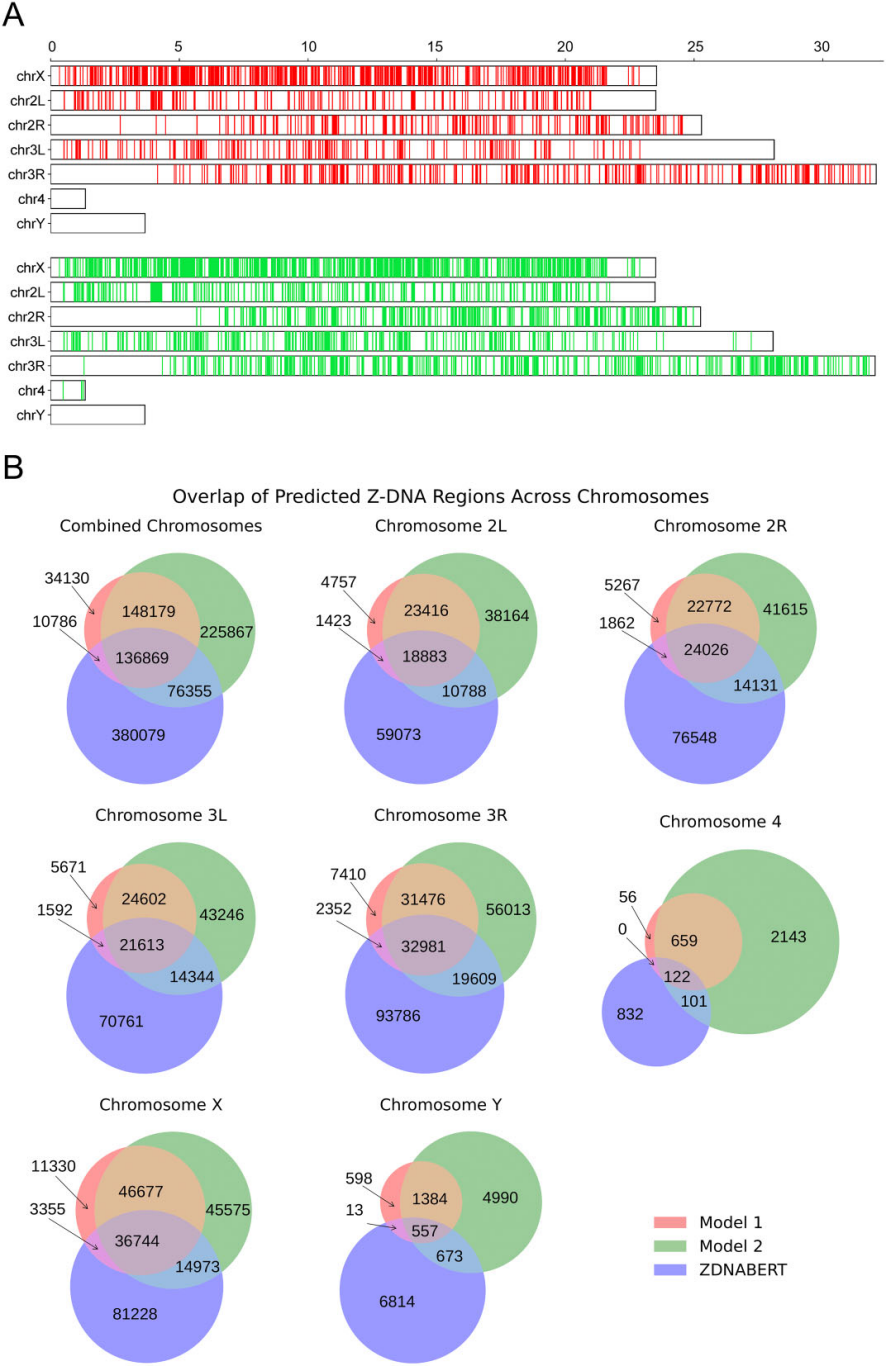
Case study of ZFS distribution in Drosophila. (**A**) Positions of long ZFS hits (24 bp and more) along *D. melanogaster* chromosomes; red bands are long ZFS predicted by Model 1 and green bands are long ZFS predicted by Model 2. Chromosome scaling is in Mbp. (**B**) Venn plot of overlaps of predicted Z-DNA forming regions (total length in bp) between the two default models of Z-DNA Hunter (Model 1 and Model 2) and Z-DNABERT.

The distribution of long ZFS (equal to or longer than 24 bp) along *Drosophila* chromosomes revealed an interesting phenomenon (Fig. 2A). First, chromosome Y did not contain any long ZFS for both Model 1 and Model 2 predictions. Also, there was a significant depletion of long ZFS in subcentromeric regions for chromosomes 2 and 3. Minichromosome 4 contains only a few long ZFS for Model 2. Finally, chromosome X contained much more long ZFS than all other chromosomes. Considering Model 2, there were 925 long ZFS on chrX and only 312 long ZFS on autosomal chr2L, which are of very similar length. Our new tool was compared with the Z-DNABERT program developed by Maria Poptsova’s group [36]. The overlaps between Z-DNA Hunter Models 1 and 2 and Z-DNABERT predictions for specific *D. melanogaster* chromosomes are illustrated in Fig. 2B. All three approaches identified a total of 136 869 bp with Z-forming potential. The observed differences, or unique hits, can primarily be attributed to the fact that Z-DNA Hunter employs a classical pattern-based approach, while Z-DNABERT is based on the transformer algorithm DNABERT [37]. Each tool has its own strengths and weaknesses regarding sensitivity and specificity for various Z-DNA-forming loci. Therefore, it may be beneficial to use them as complementary methods in future computational studies focused on Z-DNA prediction. In terms of runtime, our models complete predictions in under 0.15 s per 1 Mbp. In contrast, Z-DNABERT requires ~94–140 s per 1 Mbp on Google Colab A100 or T4 GPU, respectively. This includes postprocessing overhead caused by CUDA-to-CPU result conversion.

To evaluate the predictive performance of our tool, we analyzed a dataset of experimentally validated ZFS within the human genome from Shin *et al.* [38]. Shin *et al.* FASTA headers were first converted to 0-based half-open coordinates. Z-DNABERT and Z-DNA Hunter predictions were likewise represented as BED-like 0-based half-open intervals, and a match was defined as any overlap of ≥1 bp. Z-DNA Hunter showed good overlap with validated ZFS (up to 73% with Model 2, window size 10, and <3% false positives), especially when using shorter window lengths. Z-DNABERT achieved higher sensitivity (87.7%) but produced more false positives (11% if used in full genome analysis, but up to 62% in analysis of individual regions identified by ChIP-seq). For comparison, in the same setting (Model 2, window size 10), Z-DNA Hunter reached 72.6% sensitivity but still missed 27.4% of validated ZFSs (false negatives). A detailed summary of this benchmarking can be found in Supplementary Material S2. These results demonstrate that Z-DNA Hunter provides results with the minimum false-positive results and more precise predictions, especially in short sequence analyses.

**Figure 3.**
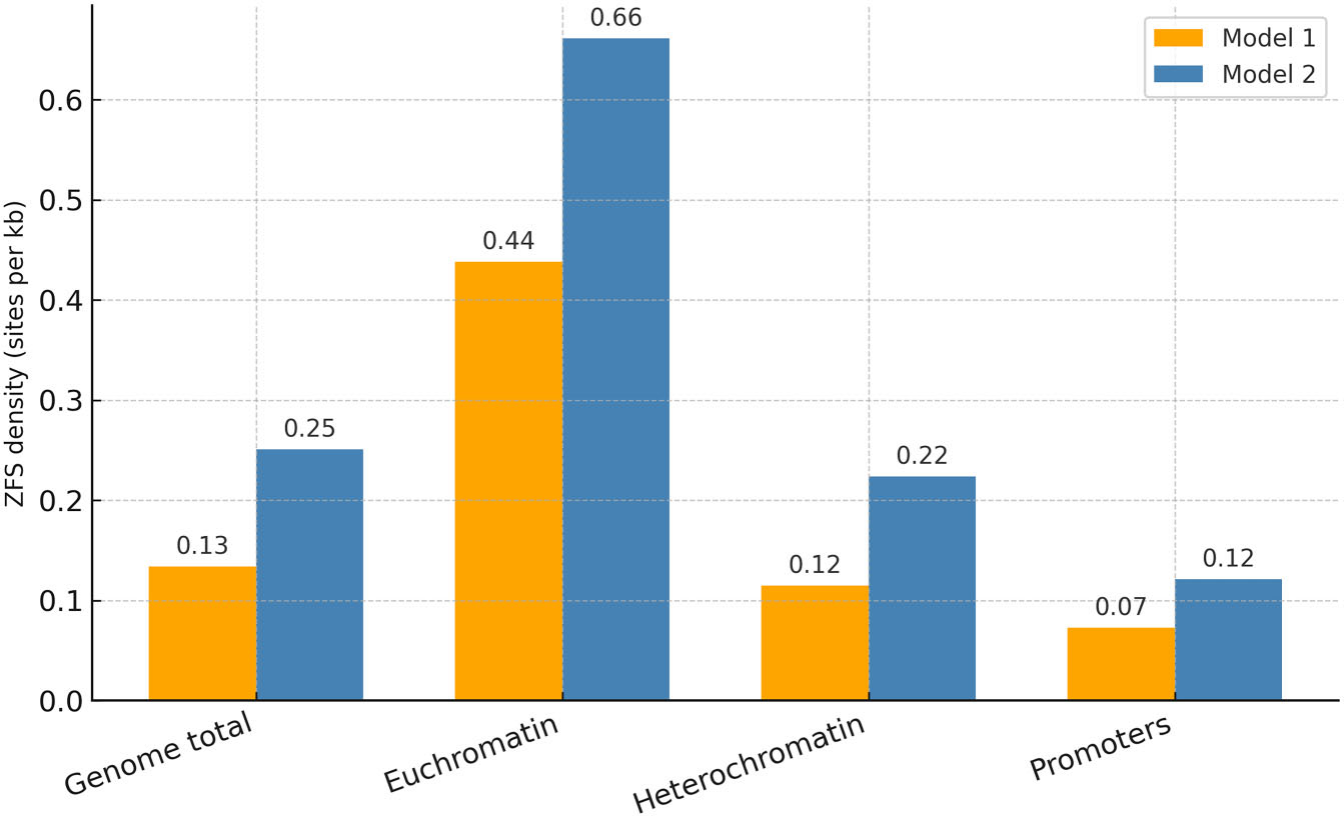
Density of predicted ZFS across genomic compartments in *D. melanogaster* (dm6). Bars show ZFS densities (sites per kb) predicted by Model 1 (orange) and Model 2 (blue) in the whole genome, euchromatin, heterochromatin, and annotated promoter regions. Both models reveal an approximately three-fold enrichment of ZFS in euchromatin relative to heterochromatin, whereas promoter cores show lower ZFS density than expected, suggesting preferential localization of ZFS to non-promoter euchromatic regions.

To characterize further the genomic context of predicted ZFS, we compared their distribution between euchromatic and heterochromatic regions. Euchromatin and heterochromatin intervals were defined using bisulphite-seq-based chromatin state annotations compiled from the ChIP-Atlas resource [39] for the *D. melanogaster* genome (dm6), yielding 8.9 Mb of euchromatin in a functional sense (6.2% of the genome) and 134.7 Mb of heterochromatin (93.7%). Note that this functional euchromatin:heterochromatin ratio differs from that estimated using cytologically defined euchromatin/heterochromatin borders in mitotic chromosomes [40] and in the release 3 (dm3) euchromatic and heterochromatic sequencing efforts [41, 42]. These studies estimate 117 Mbp of euchromatin and 59 Mbp of heterochromatin in females and a further 41 Mbp of heterochromatin in males (the 41 Mbp Y chromosome is almost entirely heterochromatic, which so far in release dm6 has meant only 3.67 Mbp have been sequenced [43]). Promoter regions (*n* = 13 400) were obtained from the EPDnew database [44] and defined as 499 bp upstream to 100 bp downstream of the annotated transcription start site. Although the majority of ZFS loci reside within heterochromatic DNA in absolute numbers, this reflects the overwhelming genomic representation of heterochromatin in our dataset derived from bisulfite sequencing. Normalization by sequence length revealed a markedly different distribution: in Model 1, ZFS occurred at a density of 0.44 sites per kb in euchromatin versus 0.12 per kb in heterochromatin, while in Model 2 the densities were 0.66 and 0.22 per kb, respectively (Fig. 3). Thus, across both models, ZFS are enriched approximately three-fold in euchromatic DNA. We next examined overlap with promoters. In Model 1, 583 ZFS intersected annotated promoters, compared with 970 in Model 2. Given that promoters occupy ∼5% of the genome, random placement would predict ∼960 and ∼1 795 overlaps, respectively. The observed counts therefore represent ∼40%–45% fewer overlaps than expected under a random model, indicating that ZFS are not preferentially positioned in promoter cores. Instead, their enrichment in euchromatic DNA suggests a bias toward regulatory neighborhoods more broadly, including gene-proximal intergenic regions and chromatin boundaries, where Z-DNA formation may influence local accessibility and transcriptional control.

## Discussion

Recently, several approaches for ZFS prediction have been developed, including Z-DNABERT [36], DeepZ [45], and ZSeeker [46]. However, these tools have some limitations. For instance, they either lack a graphical user interface and immediate availability through an online web server or they have restrictions on input file size, which prevents the analysis of entire eukaryotic chromosomes. Previously, the Z-Hunt tool—based on a thermodynamic model—was available for Z-DNA prediction [47]. Although it is no longer accessible, its results are broadly comparable to those from Z-DNA Hunter, especially for canonical motifs. The two tools differ in methodology, but both effectively identify high-propensity Z-DNA regions. Unlike previous algorithms, our implementation gives users full flexibility to adjust parameters, enabling the detection of ZFS beyond purely (GC)n-based motifs. Previous work using radiolabelled (CA)n.(GT)n DNA probes in *Drosophila* genomic DNA or polytene chromosomes showed that ~0.05% of the genome is composed of these repeats [48], with high enrichment especially within chrX, while signals were absent in chr4 and in β-heterochromatin regions [49]. The total length of (CA)n.(GT)n repeats found by Z-DNA Hunter [0.06% of total dm6 genome assembly using Model 2 with an at least two-fold enrichment on the X chromosome (0.106% of chrX sequence), compared with the autosomes] is similar to previous findings in Drosophila and its polytene chromosomes [48, 49]. While Z-DNABERT offers high sensitivity due to its deep learning architecture, its higher false-positive rate limits its precision. In contrast, Z-DNA Hunter’s pattern-based approach yields more accurate results for canonical motifs, particularly in short sequences. This makes it a more reliable tool for genome-wide screening of ZFS with minimal noise. The comparison of previously published algorithms is shown in Table 2. Our Z-DNA Hunter web tool is designed to provide a user-friendly option for researchers working on Z-DNA sequence predictions. Our goal is to promote the overall development of the field, enhance the understanding of ZFS patterns, and enable users to overlay these patterns with functional (epi)genomic regions via BedGraph outputs.

**Table 2.** The comparison of available ZFS algorithms

Feature/tool	Z-DNA Hunter	Z-Hunt	Z-DNABERT	DeepZ	ZSeeker
Web Access	Yes	No	No	No	Yes
Size Limit	Genome scale—2 GiB, server based	Not available	Based on the user computer	Based on the user computer	15 MB
BEDGRAPH Export	Yes	No	No	No	Yes
Customization	Thresholds, window size	Fixed model	Model tuning	Embedding options	Adjustable thresholds and scoring parameters
Methodology	Pattern-based	Thermodynamic	Deep learning (BERT)	Deep learning (CNN/RNN)	Scoring algorithm calibrated on experimental data
Runtime	Seconds/genome	Minutes to hours	Minutes/genome	Minutes/genome	Minutes/genome
Sensitivity	High for canonical motifs	High for thermodynamically stable motifs	High (ML benchmarked; strong recall)	Strong on ChIP-seq–validated sites	Improved over Z-Hunt on validated sites
Specificity	High for canonical motifs; limited outside motif space	High for strong thermodynamic motifs; lower for weak/short motifs	High (best ML precision–recall in benchmarks)	Moderate (improves PR over Z-Hunt but below BERT-based models)	Moderate–high (scoring optimized; better than Z-Hunt on validated sites)

The table contrasts web accessibility, input size limitations, export options, customization, methodology, runtime, and relative predictive performance across five representative tools. Abbreviations: CNN – convolutional neural network; RNN – recurrent neural network; ML – machine learning; PR – precision–recall (a performance metric comparing recall against the precision of predictions); BERT – Bidirectional Encoder Representations from Transformers, a transformer-based deep-learning architecture used for sequence modelling.

Our analysis revealed a non-random distribution of long ZFS across the *D. melanogaster* genome. The enrichment of long ZFS on the X chromosome observed in our analysis may reflect its unique regulatory roles, higher gene density, and chromatin accessibility, which are conducive to Z-DNA formation. In contrast, the absence of long ZFS on the Y chromosome aligns with its high non-dinucleotide satellite repeat content and low transcriptional activity—factors that are generally correlated with a lower presence of Z-DNA forming sites [50, 51, 52]. These findings suggest that Z-DNA Hunter can help uncover biologically relevant patterns of Z-DNA distribution, with potential implications for understanding genome architecture and regulation in cellular and medical contexts. The biological relevance of Z-DNA in *Drosophila* is further supported by early biochemical studies. Jovin *et al.* identified a Z-DNA-binding protein (Topoisomerase 2α) in *D. melanogaster* tissue culture cells and embryos, demonstrating the presence of cellular machinery capable of recognizing the left-handed DNA conformation [53]. Recent advances in the field have also highlighted the biological significance of Z-DNA and its binding proteins. Z-DNA and its RNA counterpart, Z-RNA, are increasingly recognized as dynamic regulators of gene expression, chromatin remodeling, and immune responses. A recent study identified numerous novel Z-DNA/Z-RNA binding proteins based on structural similarity to the canonical Zα domain, expanding the known repertoire of Z-DNA interactors [54]. These findings underscore the importance of accurate ZFS prediction tools in elucidating the functional roles of Z-DNA in diverse cellular contexts. Moreover, the discovery of synthetic peptides, such as KGZIP, that can specifically bind and stabilize Z-DNA structures opens new avenues for experimental validation and manipulation of Z-DNA *in vivo* [55]. These developments reinforce the need for integrative computational platforms like Z-DNA Hunter, which can facilitate hypothesis generation and guide experimental design.

In conclusion, Z-DNA Hunter represents a significant step forward in the computational analysis of Z-DNA-forming sequences. By offering a web-accessible, user-friendly interface and support for genome-scale input, it addresses key limitations of existing tools. When used in conjunction with other predictive models and experimental data, Z-DNA Hunter can contribute to a more comprehensive understanding of the structural and functional landscape of Z-DNA across the tree of life.

## Supplementary Material

lqaf166_Supplemental_Files

## Data Availability

The source code of the algorithm and web server is available at https://git.pef.mendelu.cz/bioinformatics/. Remote access at https://github.com/patrikkaura/dna-analyser-ibp. The API is freely accessible on this web page at https://bioinformatics.ibp.cz/swagger-ui/index.html. The Drosophila genomic data underlying this article (Drosophila genome dm6) are available in the NCBI Reference Genome Database at https://www.ncbi.nlm.nih.gov/datasets/genome/GCF_000001215.4/. The Z-DNA Hunter results of *D. melanogaster* genome are available at the following UCSC Genome Browser link: https://genome-euro.ucsc.edu/s/Alistair%2CCTCF/dm6%2CPetrovic%20et%20al.%2Cfinal
